# Issues Related to Late-Life Sexuality: Sex in Long-Term Care

**DOI:** 10.1093/geroni/igaa057.2030

**Published:** 2020-12-16

**Authors:** Rachael Spalding, Peter Lichtenberg

## Abstract

Despite surrounding social stigma and stereotypes of the “asexual older adult,” older adults, including those residing in long-term care facilities, indicate that expressing their sexuality continues to be important to them (Doll, 2013). This presentation will feature presentations regarding recent research and perspectives relevant to late-life sexuality with a focus on how issues of sexual expression may particularly emerge in long-term care settings. Dr. Maggie Syme will present findings from mixed-methods, consumer-based approaches that elucidate how current and future long-term care residents view late-life sexuality, with a focus on the practical applications of these findings to inform facility administration and policies. Ethical and legal issues surrounding sexuality in long-term care will be discussed by Dr. Pamela Teaster, who will present ethical models that can translate into potential best-practice recommendations and strategies. Rachael Spalding will discuss the paucity of psychometrically sound assessment tools for measuring attitudes towards late-life sexuality and discuss their development of such a measure. Finally, Dr. Lilanta Bradley and Dr. Pamela Payne-Foster will present a framework for sexual agency in late-life and identify relevant gaps in the literature regarding gender, ethnicity/race, and geographical differences. Ultimately, this presentation will offer a forum for lively discussion among attendees regarding these pertinent topics.

